# Assessing web 2D user interface experiences in mixed reality

**DOI:** 10.1016/j.heliyon.2024.e31916

**Published:** 2024-05-28

**Authors:** Yongkang Xing, Conor Fahy, Jethro Shell

**Affiliations:** a Guangdong University of Finance, Center of Experimental Teaching, Guangzhou, 510521, China; b De Montfort University, Institute of Artificial Intelligence, Leicester, LE1 9BH, UK

## Abstract

Mixed Reality (MR) technologies have the potential to revolutionize how we interact with various fields, such as medicine, education, and communication. However, existing studies have not comprehensively investigated the overall performance of 2D user interfaces (UIs) in 3D spaces. There are gaps and questions that have not been properly addressed in the transition from 2D to 3D UIs. To investigate this, we design an experiment with 80 participants to evaluate the 2D UI user experience on MR platforms. Our study reveals that compared with desktop devices, the website user experience on MR platforms leads to poorer learning performance. One-to-one interviews with participants reveal issues with both the hardware field of view and color definition, as well as the UI. Based on these findings, we propose that a generalized and optimized 3D UI would reduce control difficulties and improve the learning experience provided by MR platforms. Our study provides critical data that can be used to enhance 3D UIs on MR platforms.

## 1 Introduction

Mixed Reality (MR) combines augmented reality (AR) and virtual reality (VR) into a single concept [1]. MR devices integrate the hardware of VR with the interactive capabilities of AR, enabling users to control their experiences through gestures and voice commands using MR headsets [1], [2]. MR aims to seamlessly blend virtual objects or small environments with the real world, offering tremendous potential in diverse fields such as media, entertainment, communication, and education [3]. To address the wide range of reality-interaction technologies and their associated definitions, the term Extended Reality (XR) has been introduced, encompassing AR, MR, and VR [4]. Recent evaluations of the state-of-the-art literature have revealed that XR research is transitioning from theoretical exploration to practical applications, particularly in the simulation and education domains [5]. As a result, XR has emerged as a significant area of interest for future global research and development.

Spatial user interfaces (SUIs) could potentially play an essential role in MR by seamlessly integrating the physical and virtual worlds [6], [7]. SUIs allow users to interact with digital content and information in three dimensions, offering a level of immersion and intuitiveness that was previously unattainable [7]. This could potentially revolutionize various sectors, from creating immersive educational experiences and enhancing design and creativity to improving productivity in the workplace and enabling more engaging social interactions.

However, many existing commercial MR/VR applications retain the traditional 2D layout with limited fundamental gesture interactions in the 2D user interface (UI) panel, such as scrolling, dragging, and zooming. A typical example is the Gear VR home page [5]. It arranges the UIs in a plane, similar to traditional web/mobile design. Although the UIs are in a 3D space, all UI components are still positioned on the same plane.

Wang's research indicates that any nuances of user behavior such as language, environment and culture could affect the user experience [8]. Compared to a 2D plane, the 3D space can provide a 3D environment which is a significant difference between traditional and MR devices which could directly affect user exploration. Notably, many applications need to adapt to SUIs on MR platforms. Many researchers have begun to delve into this field. When studying the current state of the art, we find research that has focused on improving SUIs [9], [10]. The existing research focuses on replacing the existing 2D layout with a redesigned 3D layout for native/hybrid applications. However, these studies have not discussed the overall performance of 2D UIs in a 3D space. For instance, are there any remaining advantages, even minor ones, to using 2D UIs on MR platforms instead of SUIs? If 2D UIs are unsuitable for 3D spaces, what are their major weaknesses according to user feedback?

**Figure 1:**
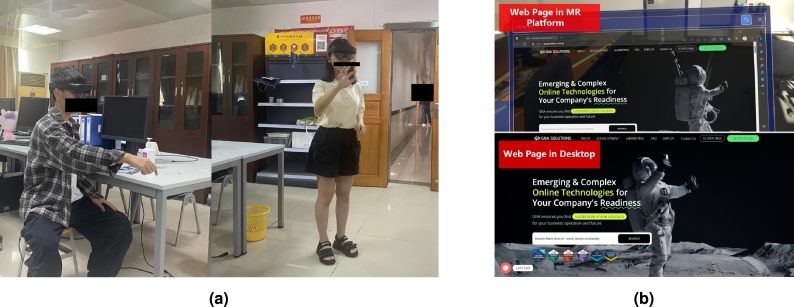
An experimental overview: (a) the HoloLens 2 group user test; (b) the website UI deployed in HoloLens 2 (MR device) and on the desktop platform.

Considering the above questions, in this work, we focus on an MR platform and conduct evaluations of user experiences while exploring 2D UIs on MR devices. We analyze user feedback on the 2D UI user experience in the 3D space. We design a comparative user experiment, as illustrated in Fig. 1a and 1b. The user experiment aims to provide crucial data references for UI researchers working on improving SUIs in MR platforms. Additionally, the spatial experience data can also benefit the VR field due to the similar spatial technical concepts. This research has the following objectives:

• To evaluate the effectiveness of 2D UIs in MR platforms through a comparative experimental design involving 80 participants.
• To evaluate the overall reading performance in the user test facilitated by a compilation of user feedback obtained via a combination of questionnaire surveys and semi-structured interviews. The aim is to identify potential issues and provide a comprehensive effectiveness evaluation through the analysis of these data.
• To analyze user data and MR interaction features. The interview analysis aims to provide insights for enhancing the user experience based on the collected data.

## 2 Background study

To conduct a comprehensive user test experiment, suitable UI cases are required. In this study, we aim to compare the user experiences provided by desktop and MR platforms. Thus, we need to identify cases that provide the same content on both desktop and MR platforms, to allow us to monitor the relative performance of the two platforms. All MR devices come equipped with a web browser that supports fundamental gesture interactions, such as scrolling, dragging, and zooming, using the default desktop version UIs [11]. Therefore, websites with satisfactory 2D UIs could serve as suitable cases for this study.

Websites have become an integral part of our daily lives, [12] and as smart devices cater to a larger consumer group, they should support web browsers that align with common usage patterns. For instance, mobile devices offer browsers and HTML5 responsive layouts, enabling users to seamlessly transition their digital experiences from desktop to mobile [13]. Over the past decade, mobile applications have become increasingly essential in our daily routines [14]. Therefore, it is crucial for MR platforms to support website exploration, expanding their applicability and meeting the expectations of potential users. In recent years, the Immersive Web Working Group under the W3C has proposed WebXR as a general standard [15]. The WebXR Device application programming interface (API) provides developers with a powerful tool to leverage the latest research outcomes, reducing development time and costs [16], [17]. By accessing XR features such as 3D rendering, position tracking, and gesture recognition, the Web XR Device API simplifies the development process, in an approach similar to that used for native applications [5], [18]. Considering the rapid growth of WebXR, this study could provide valuable reference material for web designers and developers designing web SUIs.

Before examining the user experience, it is necessary to identify typical study cases. This study begins by providing a brief overview of website classification. It explores different types of websites and their distinct features. Additionally, the specific research objects are identified and outlined based on a background study, establishing a clear focus for this study.

### 2.1 Website classification

Broadly speaking, there are two primary website types: static and dynamic [19]. Dynamic websites incorporate a back office, known as a content management system (CMS), which enables the dynamic management of commerce and online service information without the need for coding. This system empowers website providers to efficiently manage website content without requiring specific web development knowledge. Dynamic websites can be further categorized into three types, commercial (e-commerce), service, and mixed websites [20]. E-commerce websites typically operate online shopping businesses using one of three modes, business to business (B2B), business to client (B2C), and client to client (C2C) [20]. B2B websites facilitate digital commercial transactions between vendors and suppliers. B2C websites focus on providing a seamless online shopping experience for consumers. C2C websites enable small-scale commercial relationships between consumers through bidding platforms. Service websites, on the other hand, offer convenient access to various daily services without geographical or time restrictions [20]. These services may include business (e.g., banking), education, entertainment, and communication services. Lastly, mixed websites combine elements of both service and commerce, such as websites that integrate shopping and entertainment modules [20].

Compared with dynamic websites, static websites typically contain less interactive modules and do not require a back-office management system. These websites generally provide essential business information and include basic interactive elements, such as contact forms. Unlike dynamic websites, static websites do not undergo frequent updates, and their information remains relatively static without dynamic management. Despite their limited functionality, static websites comprise a significant proportion of global websites. According to web hosting statistics, static websites accounted for 51.3% of global websites in 2022 [21]. Therefore, it is crucial to include static websites in our discussion.

### 2.2 Research objects

Considering the above classification, we selected two types of websites to evaluate reading efficiency: a publication service website and a business static website.

GNA Web (GNA) (English Version: https://gnasolutions.com.au/, Chinese Version: https://gnawebdesign.com.au/) is a standard business website with general static pages [22]. These websites feature a smooth 2D UI design, and their primary goals are to attract consumers to explore and initiate a quote. They arrange key information with significant guidance throughout their websites. This is a suitable case to evaluate how consumers respond and how much information they gather from such websites. Therefore, the subsequent research uses GNA as a test sample of static websites.

CNKI (English Version: https://oversea.cnki.net/, Chinese Version: https://www.cnki.net/), is a publication website that shares the most advanced Chinese academic articles [23]. The website provides a smooth interaction experience, helping users find and read the latest research output from China. The website offers clear guidance to assist users in finding their desired reading material online. We can use this website to evaluate the overall interaction experience and learning efficiency of such websites. In the following sections, CNKI is considered the experimental example of a dynamic website.

## 3 Related work

### 3.1 Questions

Considering the above background study, we list the research questions and hypotheses in Table 1.

**Table 1:** Research Questions List.

Item	Question	Variables	Hypotheses
1	Do 2D UIs provide a better user-experience on MR platforms than in desktop computer	3D Environment and 2D Device	No
2	Do complicated interactive tasks in 2D UIs provide a worse user-experience on MR platforms?	Static Websites and Dynamic Websites	Yes
3	Any remaining minor advantages to using 2D UIs on MR platforms?	N/A	Yes (Very few, such as familiarity with 2D UIs)
4	What are the major weaknesses in 2D UIs on MR platforms according to user feedback?	N/A	Resolution, brightness, and imaging performance (Based on Previous Study)

The first question is the key research feature which was raised at the beginning of the Introduction, and we need to analyze the performance of 2D UIs (same UIs) in 3D environments and on 2D devices. Based on Wang's study, this study assumes that different environments can significantly affect the user experience [8].

Based on the previous findings, there are a lot of websites, and exploring dynamic websites can cause more complicated interactive tasks than static websites [19]. Therefore, the second question aims to find out if different difficulties in interactive tasks may cause differences in user feedback. The study expects that more complicated tasks may lead to a worse user experience with 2D UIs on MR platforms.

The third question is whether there are any remaining minor advantages to using 2D UIs on MR platforms. Pommeranz's study proves that users can prefer the things they are familiar with [24]. Thus, the research team believes that there are still some minor advantages, such as users being more familiar with using 2D UIs than SUIs, so they may prefer the traditional interactive way in 3D spaces.

Several studies have explored negative XR user experiences in various domains. For example, Zhan et al. reviewed the challenges associated with XR hardware aspects such as resolution, brightness, and imaging performance [25]. Rickel et al. conducted a user test highlighting the negative effects of typing on MR platforms [26]. Considering the above hardware limitations, we assume, in the last question, that 2D UIs may also encounter similar issues and have negative effects on MR platforms.

### 3.2 Methods

Following the above questions, the purpose of this work is to test these assumptions by conducting a focused user experiment on potential challenges. Before conducting the user experiment, a suitable user test method must be selected because there are various existing options. We refer to different VR user test projects. Lovreglio et al. conducted a comparative study to evaluate the effectiveness of VR-based learning among K-12 learners [27]. The study compared the learning outcomes of students who engaged in VR-based educational activities with those who used traditional learning methods [27]. The findings demonstrated the potential of VR in enhancing learning experiences and improving knowledge retention. Wu investigated the effectiveness of VR training and compared it with video-based training in the context of fire extinguisher skills [28]. The study compared the performance and knowledge acquisition of trainees who received VR training with those who underwent video-based training [28]. The results highlighted the advantages of VR in providing realistic and immersive training experiences, leading to improved skill acquisition and retention. Eiris et al. conducted a comparative study to analyze the effectiveness of VR-based construction safety training [29]. The study compared the safety knowledge acquisition and retention of participants who received VR training with those who underwent traditional safety training methods [29]. The findings demonstrated the effectiveness of VR in improving safety awareness and reducing workplace accidents. Janabi et al. carried out a comparative study to evaluate the effectiveness of an MR headset in minimally invasive surgery [30].

Considering the above previous projects, a comparison-based experimental method should be suitable for evaluating the effectiveness and user experience in XR fields. This approach allows for a comprehensive assessment by comparing specific conditions and analyzing the advantages and disadvantages of MR, VR, and traditional browsing tools. The first two questions have variables, so the comparison method should be used to evaluate these questions. Based on the comparative study, the study can collect enough data to analyze the other two questions.

## 4 Experiment

The subsequent sections will delve into the participants involved, the experiment conducted, and the questionnaire form distributed in this study.

### 4.1 Participants

This study aims to conduct a comparative analysis of the performance of various groups across different platforms, ensuring that the underlying conditions in each group are equivalent. Furthermore, it is imperative that the participants have experience with using online applications, to facilitate their ability to discern the disparities between MR and desktop environments. Given these requirements, (young) undergraduate students are appropriate subjects for this study [31]. Thus, 80 participants were recruited from the Guangdong area to join the user experiment. All participants were undergraduate students at Guangdong University of Finance. Table 2 shows that the participants were divided randomly into four groups named Groups A, B, C, and D. Each group had 20 participants. The experiment used different platforms and reading material to explore the web content. The platforms and reading material were the contrasting conditions in each group. We confirm that informed consent was obtained from all participants.

**Table 2:** Participant demographics.

	Group A	Group B	Group C	Group D
Participant Number	20	20	20	20
Average Age	20.3	20.1	20.0	20.15
Male:Female	1:1	1:1	1:1	1:1
Study Background	Undergraduate Student	Undergraduate Student	Undergraduate Student	Undergraduate Student
Platform	MR(HoloLens 2)	Desktop	MR(HoloLens 2)	Desktop
Reading Material	Static Website(GNA)	Static Website(GNA)	Dynamic Website(CNKI)	Dynamic Website(CNKI)

In the experiment, the exploring platforms were divided into desktop and MR platforms. For both websites, the UIs were run in desktop version on a computer. In the MR platform, the websites were also primarily built for 2D (desktop) UIs through the browser. HoloLens 2 provides the most comfortable and immersive MR experience [32]. The Edge browser is the default browser in HoloLens 2. We tested the browser and found no significant visual or interactive differences between HoloLens 2 and the desktop. Thus, we selected HoloLens 2 to test the MR devices and conducted the experiment using the Edge browser.

Groups A and B read the GNA business website (the static website) but used different platforms (condition). Groups C and D explored several articles from CNKI (the dynamic website) on different platforms. Groups A and C used the MR devices (HoloLens 2), while Groups B and D used the desktop computer. Before and after the tests, each participant completed online questionnaire forms about the website's information.

### 4.2 Questionnaire details and score calculation

The experiment contained two questionnaire forms: the user experience questionnaire (UEQ) form and the learning experience questionnaire (LEQ) form. The UEQ is a comprehensive survey for obtaining feedback from users of a software/tool with 26 indicators [33]. The UEQ covers six main scales, including attractiveness, efficiency, predictability, stimulation, transparency, and originality [33]. These are the key factors that influence users' intentions to use the technology [34]. These factors help reliably assess the software/tool for quality and user experience [35]. The study collected the UEQ data and analyzed each indicator's data using average/median and variance values. Additionally, each scale was analyzed by average value, p-value, confidence intervals, and effect sizes. LEQ aims to measure how much key information is learned from the website. The questionnaires were designed in consultation with the website owner and validated by website authors and article authors. Thus, we ensured that the questions asked pertained to important information that the authors intended for the readers or consumers to grasp. Before conducting MR experiments with Groups A and C, we tested the questionnaires on the traditional groups (B and D) to ensure the validity of the LEQ.

The static website questionnaire form included seven single-choice and three alternative questions. The form covered the history and business service details of GNA. The GNA website owner was interviewed via Skype. Table 3 presents the Group A/B questionnaire details.

**Table 3:** Group A/B questionnaire details.

Question Topic	Question Type	Question
GNA Overview Info	Single Choice	Where is GNA Based?
	Single Choice	Select the correct GNA Corporation history.

Web Design Service	Single Choice	Select the option that is outside the scope of GNA web design services.
	Single Choice	Select the correct number of GNA web design packages.
	Single Choice	Can GNA's services allow use Paypal to pay?
	Alternative Question	Do GNA offer product uploads in WooCommerce and Shopify bundles?

Web eMarketing	Single Choice	Select the correct keywords number in the Standard package of SEO services.
	Single Choice	Which service is not included in GNA's social network promotion?
	Alternative Question	Google ADS must pay to Google.

GNA Employment	Single Choice	Select the quality that GNA Corporation staff should not has.

The dynamic website questionnaire section covered the main content of two selected articles. The questionnaire had four alternative questions and six single-choice questions. The authors of the two articles were interviewed. Table 4 presents the Group C/D form details.

**Table 4:** Group C/D questionnaire details.

Articles	Question Type	Question
Article 1: Research and Practice on Wisdom Management Service of Experimentation Teaching Promoted by Big Data	Alternative Question	Does Guangdong University of Finance aim to build an applied undergraduate transformation demonstration school?
	Single Choice	Which of the following is not an advantage of the school's centralized management of experimental teaching construction?
	Single Choice	Which aspects are not included in the management framework of smart experimental teaching?
	Single Choice	Which of the following is not an effect of smart management experimental teaching?
	Alternative Question	The construction and application of the intelligent management model driven by experimental big data cannot solve the problems of the chaos of experimental teaching data and centralized management.

Article 2: Research on the Copyright Protection of Mobile Apps	Alternative Question	Is it necessary to study the copyright protection of mobile apps?
	Single Choice	Which of the following is not a concept and feature of a mobile app?
	Single Choice	What are the problems in mobile app copyright protection?
	Single Choice	In order to improve domestic mobile app copyright protection, what should not be done?
	Alternative Question	Do the administrative supervision of the relevant departments and the mobile app store have a legal arbitration institution to protect the copyright of mobile apps?

The questionnaire form had 10 points in total. Each question was worth one point. After the participants completed the online questionnaire, the scores of the different groups were calculated. For example, eleven people got six points, and nine people got three points. Each group had 20 participants so the average group score was calculated as (11 × 6 + 9 × 3)/20 = 4.65 and the median score was 6. The aim of this study was to calculate individual scores to evaluate how much key information readers/consumers absorbed after the experiment. This score was then used to evaluate the effectiveness of the experimental design in conveying key information to the users.

### 4.3 Experiment structure

**Figure 2:**
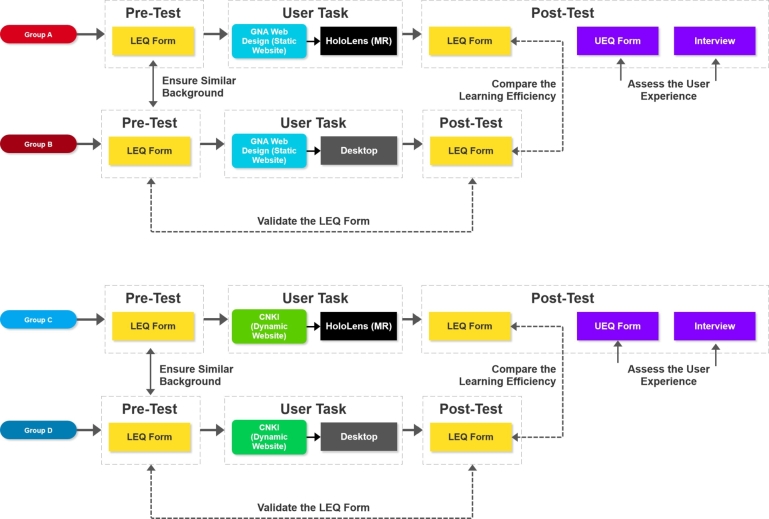
The structure of the experiment.

The experiment was divided into three sessions: pre-test, user task, and post-test, as shown in Fig. 2. To mitigate the impact of participants remembering the contents of the pre-task questionnaire while carrying out the user task, a delay was imposed between the pre-task questionnaire and the user task. If participants remembered the questions, they could have quickly located the specific pages, which would have affected the results. Thus, a time gap between the pre-test phase and the experiment was imposed of more than one month, allowing users to forget the pre-test questionnaire.

First, we had to ensure the validity of the LEQ form. Thus, Groups B and D received the LEQ form during the pre-test. After using the computer to complete the website exploration, they had five minutes to complete the LEQ form in the post-test stage. The pre-/post-test data from Groups B/D were used to validate the LEQ form and provide insights into the overall performance of the 2D UIs on the desktop.

After the LEQ form was validated, the study proceeded to the Group A/C test. Similar to the desktop groups, they received the LEQ form in the pre-test stage as well. The pre-test for Group A/C was conducted to ensure that there was no significant difference between the two groups (Group A/B, Group C/D) before the experiment. The user task and the post-test were different. Groups A and C were asked to explore the 2D UIs via HoloLens 2 (an MR device). During the user task phase, Group A participants used HoloLens 2 to explore each page from GNA Webdesign (the static website) while Group B used the desktop. The HoloLens 2 has been setup to adapt to gesture tracking and the user only can visit specific websites. Group A was required to complete the task within 20 minutes. Group C participants used HoloLens 2 to search for two specific articles from CNKI (the dynamic website) and to read them. Group C had a 30-minute time limit. The research team assisted them in operating HoloLens before the user task. After the exploration, the participants were required to complete two forms: the LEQ and UEQ forms. We used the LEQ form to compare the difference in the 2D UI learning efficiency between different devices (Group A/B, Group C/D). The goal of the UEQ form was to assess the overall user experience and satisfaction for the 2D UIs in 3D spaces (Group A/C).

Additionally, Groups A/C were required to participate in one-to-one interviews to discuss their user experiences using HoloLens 2. Each interview lasted from five to 10 minutes. The goal of the interview was to gather feedback from participants on their overall exploration experience. We intended to utilize both methods to provide valuable insights into the experiment.

### 4.4 Interview with participants

**Figure 3:**
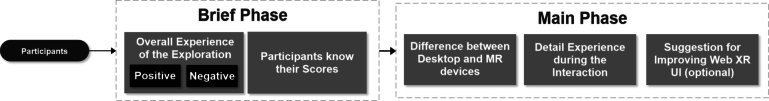
The structure of the interview.

The semi-structured interview allowed for open discussion and provided ample time for participants to provide in-depth and nuanced responses [36]. This method enabled the research team to better understand the reasons behind negative or neutral feedback [36]. Fig. 3 shows the structure of the interview. The interviews began with a brief phase during which participants could discuss their overall experience (positive and negative) during the experiments. After this, the research team provided the participants with their scores and moved on to the main phase. During the main phase, the research team engaged with the participants to explore their reading experience with the MR platform, focusing on aspects such as detailed experiences during exploration, differences between the desktop and HoloLens 2 (MR devices), and suggestions for improving the WebXR UI (optional). The research team documented the perceived shortcomings and guided the participants to identify the most significant issues.

The interview content was collected, including direct quotes from participants. The quotes were integrated into the content analysis to classify the data. The interview data were systematically summarized from the participants' responses. Based on the data, the research team could draw insights and conclusions regarding user experiences in the experiment.

### 4.5 Ethical approval

This study complied with all relevant guidelines and regulations. The protocol of this study was reviewed and approved by Guangdong University of Finance, Center of Experimental Teaching.

## 5 Results

### 5.1 LEQ form data

**Figure 4:**
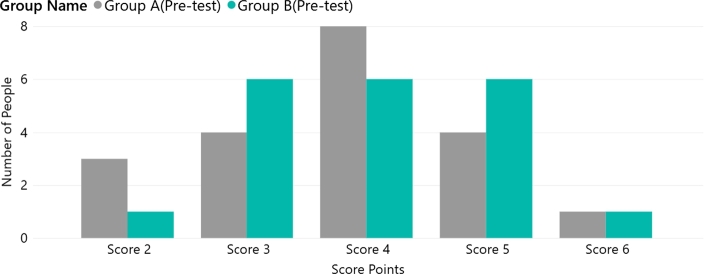
Group A/B user score distribution (pre-test).

**Figure 5:**
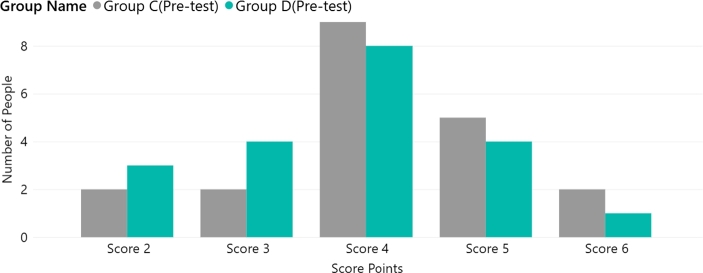
Group C/D user score distribution (pre-test).

**Figure 6:**
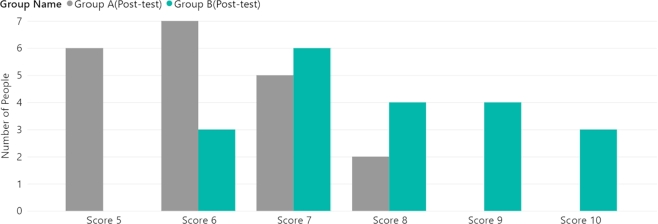
Group A/B user score distribution (post-test).

The pre-test user scores were collected for the four groups. Fig. 4 shows the user questionnaire score distribution for the static website (Groups A/B). Fig. 5 presents the Group C/D data. Table 5 shows the p-values obtained by comparing the groups. The p-value for Groups A/B was 0.557 and the p-value for Group C/D was 0.319. These p-values indicate that there was no significant difference between the two groups before the experiment. Furthermore, the median value for all groups in the pre-test stage was 4, as shown in Fig. 6. Both sets of pre-test data support that each group had similar circumstances before the experiment.

**Table 5:** The *p-value* between groups (pre-test).

Compared Group	Group A and Group B
*p-value*	0.557
Compared Group	Group C and Group D
*p-value*	0.319

**Figure 7:**
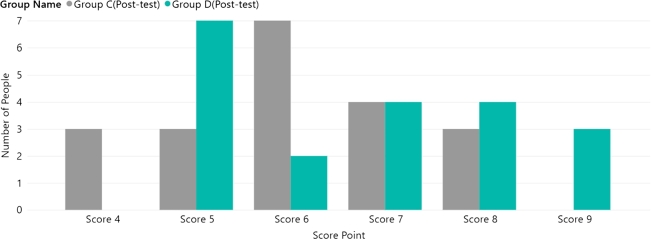
Group C/D user score distribution (post-test).

The post-test data were then collected. Fig. 6 shows a comparison of the Group A/B user score distributions. Fig. 7 shows a comparison between Groups C and D. Table 6 shows each group's average and median values in the post-test phase. The p-value for Groups A/B was 0.00003 and the p-value for Group C/D was 0.152. The p-value indicates a significant difference between Groups A/B, while Group C/D also exhibits a change compared to the pre-test. By comparing the experimental data with those of the pre-test groups, we can conclude that there are two significant trends:

• Group B's post-test average value was more than 96.49% higher than the pre-test value, while Group D's was 76.31% higher. Therefore, it can be concluded that both websites can provide convenient user experiences on desktop computers. This also validates that the LEQ form can successfully evaluate learning performance.
• Group A's post-test average data performance was 62.85% better than the pre-test performance, while Group C's was 45.78% higher. However, their performance was worse than that of Groups B and D. In other words, the 2D UIs showed poorer performance on MR devices.

**Table 6:** The average and median score data in each group.

Group Name	Group A	Group B	Group C	Group D
Average Score (Pre-test)	3.8	4.0	4.15	3.8
Median Score (Pre-test)	4.0	4.0	4.0	4.0
Average Score (Post-test)	6.15	7.9	6.05	6.7
Median Score (Post-test)	6.0	8.0	6.0	7.0

The above data show that 2D UIs on MR devices cannot provide the same learning experience as a desktop computer. The next step is to determine the reason for this result, so we turn to the UEQ form to analyze individual experiences.

### 5.2 UEQ form data

#### 5.2.1 Group A data overview

**Figure 8:**
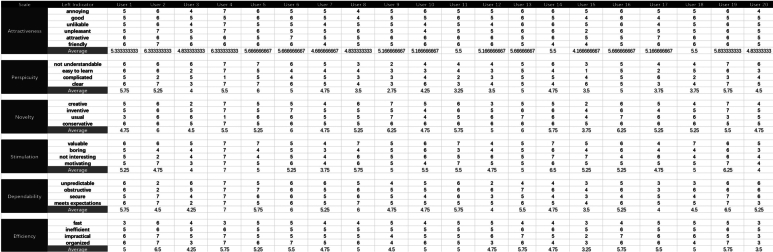
Dataset in UEQ (Group A).

The data from the UEQ form indicates that there were 22 positive indicators and four neutral indicators (Table 7, the dataset in Fig. 8). The neutral indicators included ‘Difficult to Learn,’ ‘Exciting,’ ‘Slow,’ and ‘Easy.’ Analyzing the average data, we find that the indicators ‘Difficult to Learn,’ ‘Slow,’ and ‘Easy’ are all close to the neutral point. However, the average data suggest that the ‘Exciting’ indicator leans more toward the positive side.

**Table 7:** The UEQ form data of Group A.

Scale	Left	Right	Average Value	Median Value	Variance Value
Attractiveness	annoying	enjoyable	5.3	5	0.6
	bad	good	5.2	5	0.5
	unlikable	pleasing	5.1	5	0.7
	unpleasant	pleasant	5.3	5	1.2
	unattractive	attractive	5.7	6	0.4
	unfriendly	friendly	5.5	6	0.7

Perspicuity	not understandable	understandable	5.0	5	2.1
	difficult to learn	easy to learn	4.1	4	2.4
	complicated	easy	3.8	4	1.9
	confusing	clear	5.0	5	2.1
				
Novelty	dull	creative	4.9	5	2.2
	conventional	inventive	5.4	5	0.9
	usual	leading edge	5.2	6	2.4
	conservative	innovative	5.7	6	0.3
				
Stimulation	inferior	valuable	5.7	6	1.2
	boring	exciting	4.6	4	1.3
	not interesting	interesting	5.1	5	1.7
	demotivating	motivating	5.3	5	1.3
				
Dependability	unpredictable	predictable	4.7	5	2.2
	obstructive	supportive	5.5	6	1.6
	not secure	secure	5.1	5	1.6
	does not meet expectations	meets expectations	5.3	5	1.7
				
Efficiency	slow	fast	4.3	4	0.7
	inefficient	efficient	5.3	5	0.2
	impractical	practical	5.3	5	1.7
	cluttered	organized	5.2	6	2.2

The variance values are analyzed. Nine indicators fall in the range of 0 to 0.9, 10 indicators range from 1.0 to 1.9, and seven indicators exceed 2.0. The indicators with values above 2.0 include ‘Understandable,’ ‘Dull,’ ‘Difficult to Learn,’ ‘Predictable,’ ‘Leading Edge,’ ‘Confusing,’ and ‘Cluttered.’

#### 5.2.2 Group C data overview

**Figure 9:**
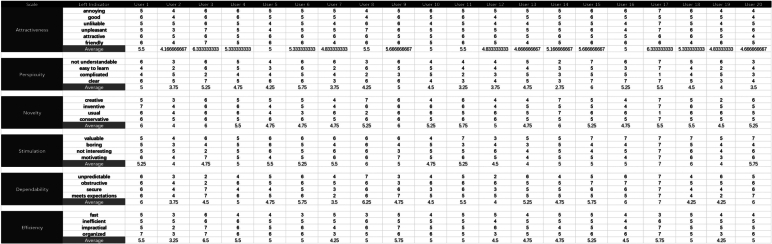
Dataset in UEQ (Group C).

In comparison to Group A, Group C's UEQ form data reveals a similar situation (Table 8, the dataset in Fig. 9). The median values show that there were 22 positive indicators and four neutral indicators, namely ‘Difficult to Learn,’ ‘Exciting,’ ‘Slow,’ and ‘Easy,’ which align with those observed in Group A. The average data also suggest that these four indicators are close to the neutral point. However, it's noteworthy that the average data for Group C are worse than those for Group A.

**Table 8:** The UEQ form data of Group C.

Scale	Left	Right	Average Value	Median Value	Variance Value
Attractiveness	annoying	enjoyable	5.2	5	0.7
	bad	good	5.1	5	0.5
	unlikable	pleasing	4.9	5	0.7
	unpleasant	pleasant	5.3	5	1.1
	unattractive	attractive	5.5	6	0.4
	unfriendly	friendly	5.4	6	0.8

Perspicuity	not understandable	understandable	4.9	5	2.1
	difficult to learn	easy to learn	4.2	4	2.1
	complicated	easy	3.7	4	1.6
	confusing	clear	5.1	5	2.1
				
Novelty	dull	creative	5.0	5	1.8
	conventional	inventive	5.3	5	0.9
	usual	leading edge	5.1	6	2.4
	conservative	innovative	5.5	5	0.4
				
Stimulation	inferior	valuable	5.7	6	1.4
	boring	exciting	4.5	4	1.1
	not interesting	interesting	5.0	5	1.4
	demotivating	motivating	5.3	5	1.3
				
Dependability	unpredictable	predictable	4.7	5	2.2
	obstructive	supportive	5.4	6	1.6
	not secure	secure	5.0	5	1.7
	does not meet expectations	meets expectations	5.2	5	2.1
				
Efficiency	slow	fast	4.3	4	0.7
	inefficient	efficient	5.1	5	0.3
	impractical	practical	5.2	5	1.4
	cluttered	organized	5.3	6	1.8

The variance values are analyzed. Nine indicators fall in the range of 0 to 0.9, 11 indicators range from 1.0 to 1.9, and six indicators exceed 2.0. Notably, the indicators with values above 2.0 include ‘Understandable,’ ‘Difficult to Learn,’ ‘Predictable,’ ‘Leading Edge,’ ‘Confusing,’ and ‘Does not meet expectation.’

#### 5.2.3 Comparison of UEQ scales between groups

Table 9 presents the average values for six scales in both Group A and Group C. The average values for all scales in both groups were above 4.0, indicating a positive user experience. In Group A, the highest average value was observed for Attractiveness (5.4), followed by Novelty (5.3), Stimulation (5.2), Dependability (5.2), Efficiency (5.0), and Perspicuity (4.5). Similarly, in Group C, the highest average value was observed for Attractiveness (5.2), followed by Novelty (5.2), Stimulation (5.1), Dependability (5.1), Efficiency (5.0), and Perspicuity (4.5). The average values for Group C were slightly lower than those for Group A across all scales, except for Perspicuity and Efficiency, which had the same average values in these groups.

**Table 9:** UEQ Scale Averages.

Scale	Group A	Group C
Attractiveness	5.4	5.2
Perspicuity	4.5	4.5
Novelty	5.3	5.2
Stimulation	5.2	5.1
Dependability	5.2	5.1
Efficiency	5.0	5.0

The small differences in average values between the two groups suggest that the user experience was relatively similar for these groups. To validate this finding, further data were analyzed to compare these two groups in Table 10. The p-values indicate that there were no significant differences between Group A and Group C in any of the UEQ scales (all p-values > 0.05). According to Cohen's guidelines, effect sizes under 0.2 are considered small, while those between 0.2 and 0.5 are considered medium [37]. Effect sizes calculated using Cohen's d range from 0.055 to 0.218, indicating small to negligible differences between the groups, with the exception of ‘Attractiveness’, which falls into the lower end of the medium range. These findings suggest that the user experience, as measured by the UEQ, was similar for these groups.

**Table 10:** Comparison of UEQ Scales between Groups.

Scale	*p-value*	Cohen's d	95% CI(Group A)	95% CI(Group C)
Attractiveness	0.495	0.218	[5.1-5.6]	[5.0-5.5]
Perspicuity	0.862	0.055	[4.0-4.9]	[4.0-4.8]
Novelty	0.654	0.143	[5.0-5.6]	[5.0-5.5]
Stimulation	0.838	0.065	[4.8-5.6]	[4.8-5.5]
Dependability	0.797	0.082	[4.7-5.6]	[4.6-5.5]
Efficiency	0.829	0.069	[4.7-5.4]	[4.7-5.3]

The results of these tests, along with 95% confidence intervals (CI) for the two groups, are presented in Table 10. The 95% CI for each scale in these groups largely overlap, further supporting the lack of significant differences. However, Group C's CI data show that the upper bound in ‘Attractiveness,’ ‘Perspicuity,’ ‘Novelty,’ ‘Stimulation,’ ‘Dependability,’ and ‘Efficiency’ is slightly lower than Group A. At the same time, the lower bound in ‘Attractiveness’ and ‘Dependability’ is slightly lower than Group A. The CI data support the average data, indicating that Group C is slightly lower than Group A.

#### 5.2.4 Data brief

The UEQ form data for Group A and C indicate that MR devices can generate a positive user experience, with 22 out of 26 indicators being positive and only four indicators (Difficult to Learn, Exciting, Slow, and Easy) being neutral. However, the variance analysis reveals high variance values (above 2.0) for several indicators within each group, such as ‘Understandable,’ ‘Difficult to Learn,’ ‘Predictable,’ ‘Leading Edge,’ and ‘Confusing.’ This suggests that users within the same group had significantly different experiences, particularly related to learning and understanding the MR device.

The comparison of UEQ scales between Groups A and C indicates that the two groups had similar user feedback, but the average values for Group C were slightly lower across most scales. Further analysis, including p-values, confidence intervals, and effect sizes, validates this finding, with no significant differences between the groups (all p-values > 0.05) and small to negligible effect sizes (0.055 to 0.218), except for attractiveness, which falls into the lower end of the medium range. The 95% CI for each scale largely overlap, further supporting the lack of significant differences, although Group C's CI data show slightly lower upper and lower bounds for some scales. The UEQ data shows that 2D UIs in dynamic websites have slightly poorer performance than static websites although the overall experience is similar. More complicated interactive tasks caused a slightly negative experience while using 2D UIs in MR platforms.

To identify the specific issues related to learning and user experience, further analysis of the interview data is necessary.

### 5.3 Interview data

Forty participants were interviewed in total from Groups A and C. Each interview started after the participant completed their exploration and questionnaire. In the interview, participants provided valuable feedback with detailed descriptions of their negative experiences with navigating websites in MR.

Initially, all 40 (100%) participants agreed that the MR device provided an interesting experience that they had not experienced on desktop or mobile devices. The first impression of MR was the main reason for leaving any positive feedback on the UEQ form. However, all participants also noted that the field of view was too limited. When participants opened multiple windows, they often lost visibility in some panels. Secondly, 37 (92.5%) of the participants said that the blurred rainbow-colored light flare affected their vision and caused visual fatigue. Thirdly, 35 (87.5%) of the participants felt tired when using the gestures for too long. Fourthly, 32 (80%) of the participants frequently made incorrect selections due to the high gesture sensitivity. Last but not least, 29 (72.5%) of the participants mentioned weak button-touching feedback.

Apart from the above hardware issues, the study asked the participants about differences between using HoloLens 2 and a desktop to explore websites. The participants put forward the following issues in HoloLens 2: the navigation buttons are too small to select; the font is too small to read; typing is tricky; and they felt tired after typing for a long time.

The interview allowed participants to provide suggestions for improving the WebXR experience. Fifteen (37.5%) of the participants provided the following primary suggestions: the navigation menu should be designed with suitable padding space for the 3D environment; each piece of content should have summary information to prompt users to explore; the content should be displayed as different sessions in the 3D space.

## 6 Discussion

This study has collected data and interview content to analyze the 2D UI user experience on MR platforms. Interview data revealed that the positive user feedback from the UEQ was due to the novelty of using HoloLens, but the 2D UIs did not make any positive impression, which matched the first hypothesis and proved that the third hypothesis - that 2D UIs have advantages in a 3D environment - is wrong. The UEQ data shows that 2D UIs in dynamic websites have slightly poorer performance than static websites, which matches the second hypothesis. However, the overall experience is similar. Furthermore, hardware and software limitations damaged the initial positive impressions of using MR devices. The challenges can be divided into two aspects: hardware and software.

The hardware of MR systems currently faces two primary issues: a blurred rainbow-colored light flare and a limited field of view (FOV), both of which, negatively impact the user experience. FOV issues have been a challenge for MR platforms for a long time, with hardware developers gradually upgrading the angles from 30 degrees in HoloLens 1 to around 50 degrees in HoloLens 2 and Magic Leap One [38], [39]. However, this field of view is still a significant weakness as the human eye horizon FOV is 135 degrees [40]. Another major issue in MR platforms, is color flaws, which become particularly pronounced in robust environmental lighting conditions and have been reported by some HoloLens users [25], [41]. Ultimately, due to material limitations, FOV and color flaws remain the current major hardware challenges in MR systems.

From the UI perspective, there are two primary directions: system interaction settings and Web UIs. The HoloLens 2 and other VR/MR devices provide a general mid-air keyboard for typing [26]. However, typing in mid-air can cause fatigue over long periods due to the lack of tactile feedback. This typing fatigue presents a critical challenge for MR platforms. While voice recognition can be used as an alternative to typing, it is not suitable for long-form text input as prolonged speaking also leads to fatigue. As a result, there is a need for innovative typing methodologies using gesture interaction to improve the user experience in MR platforms.

The hardware issues match our last hypothesis, but the UIs issues are additional findings. In contrast to the difficulties with hardware, designing improved SUIs in MR platforms could potentially solve most negative feedback related to the Web UI. By analyzing the interview data, we have summarized three primary features for improvement: navigation, content layout, and guidance. This study proposes the following possible solutions. First, an innovative navigation UI with suitable padding space is required. It should provide a convenient method to select items and sub-items with gesture interaction. Secondly, a 3D layout to display content that utilizes the MR spatial features could maximize the immersive experience. The layout could scale the selected content, to avoid font size issues. Finally, the UIs should contain clear guidance (text or icons) to help the users explore different content without distraction from various other elements in the 3D space.

Considering the above-mentioned possible solutions, it is necessary to establish a general UI framework principle. Furthermore, researchers should refer to the existing general principles of various websites and design the content display for 3D features appropriately. We believe that this could help users to easily use the application, and enhance their learning.

## 7 Conclusion

This study has found that various MR applications still use 2D UIs. We raised the question, of whether the current display mode may negatively affect exploration. To test our theory, a user test experiment was carried out with 80 participants covering static and dynamic websites. The data confirmed the primary assumption that the learning performance of 2D UIs in MR is worse than on a desktop. The study aimed to identify the primary reasons for this, and conducted questionnaires and one-to-one interviews. User feedback data showed that 2D UIs limit user learning performance in MR. More complicated interactive tasks caused a slightly negative experience while using 2D UIs in MR platforms. Both hardware and software aspects should be improved to accommodate XR features. These findings are supported by prior research, which found that the hardware limitations of MR devices can have several negative effects [25], [26].

Based on these evaluations, we believe an innovative SUI principle is necessary. It could be an immersive exploration tool for various websites and expand the user market. Unlike previous studies focusing solely on designing new 3D layouts, our research has delved into the underlying issues associated with 2D UIs on MR platforms. This study's contributions to the SUI field are threefold: firstly, it is the first large-scale experiment to evaluate the user experience of 2D UIs on MR platforms; secondly, it provides critical user test data for UI researchers to improve SUIs on MR platforms, and the VR platform can also benefit from the study due to its spatial features; and lastly, the interview analysis offers insights that could be used to enhance the user experience in SUIs.

## 8 Limitations and future directions

Although this study has identified MR UI challenges, there are some limitations to consider. Firstly, the investigation only covered two websites. Additionally, due to time and resource constraints, the questionnaire was not tested with a small group before the experiment was conducted with four groups. The test group was also restricted to young participants. Furthermore, the study did not evaluate the user experience of a smartphone layout in MR. Therefore, future research should aim to expand the scope of the websites examined and conduct a pilot test of the questionnaire with a small group before conducting experiments with multiple groups. Future work should also suggest and evaluate practical solutions, to the identified limitations of current 2D UI performance on XR platforms.

## CRediT authorship contribution statement

**Yongkang Xing:** Writing – review & editing, Writing – original draft, Methodology, Investigation, Funding acquisition, Formal analysis. **Conor Fahy:** Writing – review & editing, Writing – original draft. **Jethro Shell:** Writing – review & editing, Writing – original draft, Supervision, Methodology.

## Declaration of Competing Interest

The authors declare the following financial interests/personal relationships which may be considered as potential competing interests: Jethro Shell reports financial support was provided by De Montfort University Open Access Fund. Yongkang Xing reports financial support was provided by 14th Five-Year Planning Project for the Development of Philosophy and Social Sciences in Guangzhou (Grant No. 2022GZGJ290). Yongkang Xing reports financial support was provided by Philosophy and Social Sciences 2023 Annual Planning Project in Qingyuan (Grant no. QYSK2023055). Yongkang Xing reports financial support was provided by Philosophy and Social Sciences 2024 Annual Planning Project in Foshan (Grant no. 2024-ZDB08). If there are other authors, they declare that they have no known competing financial interests or personal relationships that could have appeared to influence the work reported in this paper.

## Data Availability

The datasets used and analyzed during the current study are available from the corresponding author upon reasonable request.

## Appendix A

In this appendix, we present Figure 8, Figure 9.
